# Hfq stimulates the activity of the CCA-adding enzyme

**DOI:** 10.1186/1471-2199-8-92

**Published:** 2007-10-18

**Authors:** Marion Scheibe, Sonja Bonin, Eliane Hajnsdorf, Heike Betat, Mario Mörl

**Affiliations:** 1 Institute for Biochemistry, University of Leipzig, Brüderstr. 34, 04103 Leipzig, Germany; 2 UPR 9073 CNRS conventionnée avec l'Université Paris 7-Denis Diderot, Institut de Biologie Physico-Chimique, 13 rue P et M Curie, 75005 Paris, France

## Abstract

**Background:**

The bacterial Sm-like protein Hfq is known as an important regulator involved in many reactions of RNA metabolism. A prominent function of Hfq is the stimulation of RNA polyadenylation catalyzed by *E. coli *poly(A) polymerase I (PAP). As a member of the nucleotidyltransferase superfamily, this enzyme shares a high sequence similarity with an other representative of this family, the tRNA nucleotidyltransferase that synthesizes the 3'-terminal sequence C-C-A to all tRNAs (CCA-adding enzyme). Therefore, it was assumed that Hfq might not only influence the poly(A) polymerase in its specific activity, but also other, similar enzymes like the CCA-adding enzyme.

**Results:**

Based on the close evolutionary relation of these two nucleotidyltransferases, it was tested whether Hfq is a specific modulator acting exclusively on PAP or whether it also influences the activity of the CCA-adding enzyme. The obtained data indicate that the reaction catalyzed by this enzyme is substantially accelerated in the presence of Hfq. Furthermore, Hfq binds specifically to tRNA transcripts, which seems to be the prerequisite for the observed effect on CCA-addition.

**Conclusion:**

The increase of the CCA-addition in the presence of Hfq suggests that this protein acts as a stimulating factor not only for PAP, but also for the CCA-adding enzyme. In both cases, Hfq interacts with RNA substrates, while a direct binding to the corresponding enzymes was not demonstrated up to now (although experimental data indicate a possible interaction of PAP and Hfq). So far, the basic principle of these stimulatory effects is not clear yet. In case of the CCA-adding enzyme, however, the presented data indicate that the complex between Hfq and tRNA substrate might enhance the product release from the enzyme.

## Background

Originally discovered as a host factor required for replication of phage Qβ RNA [1], Hfq (also named Host Factor I) has emerged as a multifunctional regulator with a variety of different targets in the bacterial cell. It forms a highly stable hexameric ring-shaped structure of identical subunits (11.2 kDa) that can polymerize as helical fibers [2]. As a member of the Sm/Lsm protein family that is involved in RNA metabolism, Hfq contains an N-terminal α-helical domain followed by an antiparallel five-stranded beta-sheet, which is the hallmark of the so-called Sm domain [3]. By its interaction with RNA, it controls the expression of many genes, stimulates the degradation of various mRNAs and modulates RNA processing events [4,5]. Its chaperone activity plays an important role for interactions between regulatory small non-coding RNAs (sncRNA) and their target messenger RNAs [6-10]. Hfq preferentially binds to A/U- rich single stranded regions, especially to poly(A) tails and U-rich elements close to hairpin structures, where it induces structural changes [11]. While the major part of Hfq is found in the cytoplasm, a certain amount binds to genomic DNA and is involved in nucleoid formation [12,13].

Furthermore, Hfq stimulates the addition of poly(A) tails to mRNAs, where experimental data indicate that it interacts with RNA and possibly also with PAP [14,15]. As a member of the nucleotidyltransferase family, this polymerase is closely related to tRNA nucleotidyltransferase (CCA-adding enzyme), an enzyme that catalyzes the addition of the CCA terminus to tRNA 3'-ends [16,17]. Here, it is demonstrated that these two enzymes do not only share structural as well as functional elements [18,19], but are also both modulated in their activity by Hfq.

## Results

### Hfq stimulates CCA-addition

**Figure 1:**
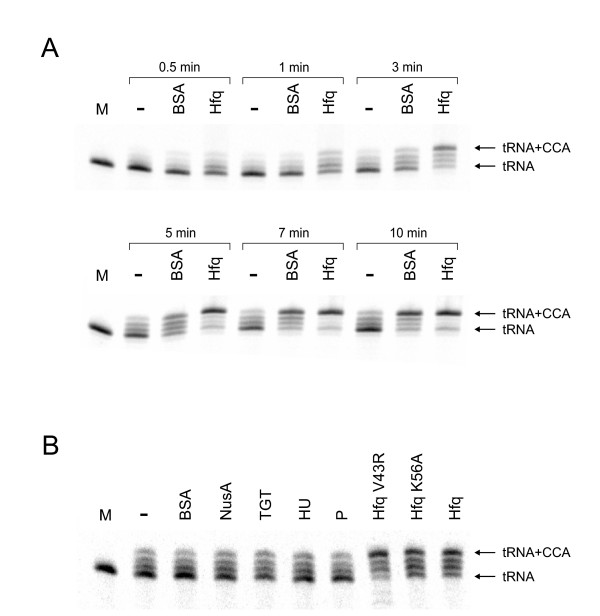
**CCA-addition is stimulated by Hfq**. **(A) **The *E. coli *CCA-adding enzyme was incubated for indicated times with radioactively labeled yeast tRNA^Phe ^without CCA-end as a substrate in the absence or presence of Hfq or BSA, respectively. The reaction products were separated by denaturing polyacrylamide gel electrophoresis. CCA-addition leads to a reduced electrophoretic mobility of the labeled tRNA, and the corresponding signal intensities indicate a dramatic enhancement of the CCA incorporation in the presence of Hfq, while the CCA synthesis without Hfq or BSA addition was only moderate. BSA also led to a considerable stimulation, probably by stabilizing the active CCA-adding enzyme. These results were verified using different tRNA substrates (*E. coli *tRNA^Ala^, phage T5 tRNA^Cys^, not shown). M, mock incubation without addition of CCA-adding enzyme; -, activity of CCA-adding enzyme without any additional protein. **(B) **CCA-addition in the presence of several RNA binding proteins, BSA, Hfq or Hfq variants. Only Hfq and the two variants V43R and K56A lead to a strong increase in CCA-addition, while all other RNA binding proteins show a much weaker stimulating effect, indistinguishable to that of BSA. NusA: transcription elongation factor (*E. coli*); TGT: tRNA guanine transglycosylase (*Z. mobilis*); HU: histone-like protein that also interacts with RNA (*E. coli*); P: RNase P protein subunit (*E. coli*).

To investigate whether Hfq has an effect on the catalytic activity of the *E. coli *CCA-adding enzyme, the recombinant enzyme was incubated at 30°C for up to 10 minutes in the presence of NTPs and Hfq. As a radioactively labeled substrate for CCA-addition, yeast tRNA^Phe ^lacking the CCA terminus was used. This is one of the best characterized tRNA molecules, and the corresponding unmodified *in vitro *transcript has a three-dimensional structure very similar to that of the fully modified *in vivo *molecule [20-23]. It is therefore an ideal substrate for *in vitro *tRNA processing and aminoacylation reactions [24-26]. In control experiments, addition of Hfq was omitted or replaced by bovine serum albumin (BSA), which is frequently used to stabilize purified enzymes in an active conformation. Furthermore, additional prokaryotic RNA binding proteins (*E. coli *NusA [27], *Z. mobilis *tRNA guanine transglycosilase (TGT) [28], *E. coli *RNase P protein [29] and *E. coli *HU [30,31]) as well as two variants of Hfq were used as controls. Hfq K56A, located in the highly conserved cavity of the hexamer, interferes with binding of the small regulatory RNA *DsrA *[32]. Hfq V43R, on the other hand, affects binding of polyadenylated *rpsO *mRNA and stimulation of PAP [33]. The reaction products were separated on polyacrylamide gels and visualized by autoradiography. As shown in Fig. 1A, CCA-addition leads to new tRNA species with reduced electrophoretic mobility, where the uppermost signal corresponds to the tRNA carrying a complete CCA terminus, while the other products indicate partial CCA-addition (incorporation of one or two C residues, respectively). While this activity is found in all assays, the time course indicates that Hfq enhances the reaction efficiency substantially. After 3 minutes of incubation, a strong increase of the reaction product (tRNA^Phe ^with CCA terminus; Fig. 1A) can be seen in the reaction assay including Hfq, while the presence of BSA leads only to a moderate product increase relative to the reaction catalyzed by the CCA-adding enzyme alone. At longer incubation times, the reactions follow this trend. However, this stimulation is not the result of an Hfq- or BSA-catalyzed nucleotide incorporation, since incubation of tRNA substrate with NTPs and Hfq or BSA alone does not lead to any detectable reaction product (data not shown). The results were verified in 8 independent experiments. Reproductions of this assay with other tRNA transcripts without CCA end (*E. coli *tRNA^Ala^, phage T5 tRNA^Cys^) further corroborated the general Hfq-mediated enhancement of CCA-addition (data not shown). The additional control experiments with RNA binding proteins and Hfq variants shown in Fig. 1B support these findings. None of the RNA binding proteins shows a stimulation of CCA-addition comparable to that of Hfq, indicating that this enhancement is indeed a specific effect of this protein. Furthermore, the fact that both Hfq variants stimulate CCA-addition comparable to the wild type protein demonstrates that this effect is different from PAP stimulation or interaction with small regulatory RNAs, where these point mutations lead to reduced efficiencies [32,33].

### Interaction of Hfq with tRNA substrate

**Figure 2:**
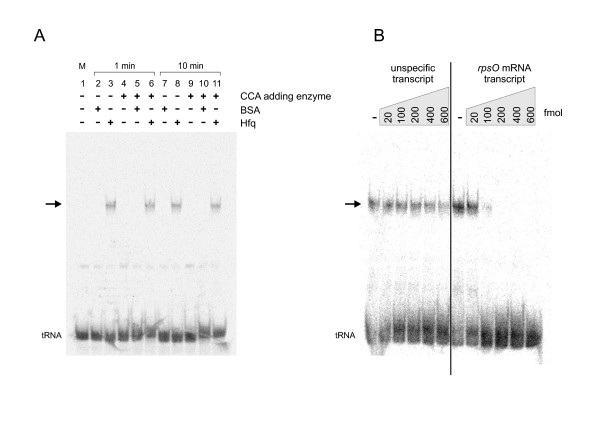
**Gel shift experiments with CCA-adding enzyme, Hfq and BSA**. **(A) **Only Hfq (either alone or in the presence of CCA-adding enzyme) was binding to the radioactively labeled tRNA substrate (without CCA terminus), leading to a reduced electrophoretic mobility on a native polyacrylamide gel (arrow). The identical band shifts in the gel after sample preincubation for 1 and 10 minutes indicate that the binding equilibrium was reached within 1 minute and that the Hfq-tRNA interaction is rather stable over time. **(B) **The tRNA/Hfq complex was competed by a nonspecific plasmid run-off transcript (left) or a transcript corresponding to the 3'-end of *rpsO *mRNA. While the *rpsO *RNA (carrying an Hfq binding site) efficiently replaced the bound tRNA at concentrations above 100 fmol (> 5-fold excess), the plasmid transcript could not compete for binding at any concentration, indicating a specific interaction of Hfq with the tRNA.

While Hfq visibly stimulates the CCA-addition, it was not clear whether this enhancement was based on an interaction of Hfq with the CCA-adding enzyme or with the tRNA substrate. Therefore, gel shift experiments with radioactively labeled tRNA and Hfq were performed under conditions identical to those of the CCA-addition. 7.5 pmol Hfq and 5 pmol ^32^P-labeled tRNA substrate without CCA-end were incubated for 1 and 10 minutes. Subsequently, samples were separated on a non-denaturing polyacrylamide gel and visualized by autoradiography (Fig. 2A). Neither BSA nor the CCA-adding enzyme (or a combination of both) lead to a shifted signal position of the tRNA, indicating that these proteins do not bind stably to the tRNA substrate. In contrast, Hfq alone as well as in combination with CCA-adding enzyme binds to tRNA and leads to a shifted migration position of the transcript. Again, other tRNA transcripts lacking the CCA terminus (*E. coli *tRNA^Ala^, phage T5 tRNA^Cys^) led to identical results, indicating that the observed binding of Hfq is not restricted to one type of tRNA molecule (data not shown). In a competition experiment, a nonspecific *in vitro *run-off transcript corresponding to part of the pCR 2.1 TOPO vector sequence could not compete for Hfq binding at any tested concentration. However, a 5- to 30-fold excess of a transcript representing the 3'-terminal sequence of the *E. coli rpsO *mRNA, a well characterized Hfq interaction partner [14], could efficiently replace the tRNA in the binding assay (Fig. 2B). These data clearly indicate that the binding of Hfq to the tRNA transcript is specific and does not represent an *in vitro *artifact. Therefore, it is likely that the Hfq-tRNA interaction is the basis for an enhanced CCA-addition in the presence of Hfq, while the stimulatory effect of BSA is probably unspecific and based on stabilization of the active conformation of the enzyme (this BSA effect is observed for many enzymes like restriction endonucleases and others). The Hfq-tRNA complex was also observed at varying concentrations of Hfq and tRNA (125 fmol tRNA and 2.5 pmol or 6.25 pmol Hfq, corresponding to a tRNA-Hfq ratio of 1:20 and 1:50, respectively (calculated for the monomer; data not shown). These ratios correspond to RNA-Hfq concentrations used in other gel shift experiments [34,35] and indicate that the interaction with tRNA molecules is stable over a range of varying concentrations of RNA and Hfq.

Interestingly, the observed interaction of Hfq with tRNA is not influenced by the presence or absence of a complete or partial CCA-terminus, since control gel shift experiments with tRNA^Phe ^ending with C--, CC- or CCA showed that all transcript versions were bound with similar affinities (data not shown). This is in good agreement with the fact that the primary task of the CCA-adding enzyme in *E. coli *is the repair of such partial CCA termini, indicating a function of the Hfq-tRNA complex in the restoration of CCA termini.

### Kinetic analysis

**Figure 3:**
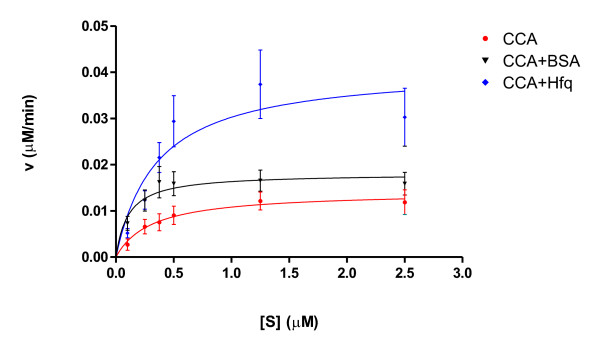
**Apparent kinetic parameters of CCA-addition**. Increasing amounts of unlabeled tRNA^Phe ^were incubated with CCA-adding enzyme, NTPs and α-^32^P-ATP in presence (CCA+Hfq (blue diamonds), CCA+BSA (black triangles)) or absence (CCA, red bullets) of Hfq or BSA. Reaction products were separated by PAGE and analyzed by autoradiography. The corresponding reaction velocities were determined using GraphPadPrism software.

To investigate the stimulating effect of Hfq on CCA-addition in more detail, Michaelis-Menten kinetic analyses were performed (Fig. 3, Table 1). In five independent experiments, apparent K_M _and V_max _values were determined in presence and absence of Hfq or BSA, respectively. Concentration of tRNA^Phe ^without CCA end was ranging from 0.1 μM to 2.5 μM. Reaction products were separated by PAGE, and the signal intensities were quantified. While the presence of Hfq did not change K_M _values compared to reaction of the CCA-adding enzyme alone (0.36 μM versus 0.31 μM), it increases the maximum reaction rate of CCA-addition from 14.16 nM/min to 41.00 nM/min (Table 1). On the other hand, the stimulating effect of BSA had only a slight impact on V_max_, but lowered K_M_. This stimulating effect can be observed particularly at very low substrate concentrations (0.01 μM), where it seems to be equivalent to the Hfq-based reaction enhancement. At higher tRNA concentrations, however, BSA leads only to a slight increase in reaction velocity compared to the reaction in the presence of Hfq (Fig. 3).

**Table 1:** Kinetic analysis of CCA-addition in presence or absence of Hfq or BSA. Apparent kinetic parameters of five independent experiments were determined and calculated in a nonlinear regression fit by using GraphPadPrism

Enzyme	Addition	K_M _(μM)	V_max _(nM/min)
CCA-adding enzyme	-	0.31 ± 0.15	14.16 ± 2.16
CCA-adding enzyme	BSA	0.11 ± 0.06	18.07 ± 2.01
CCA-adding enzyme	Hfq	0.36 ± 0.17	41.00 ± 6.52

## Discussion

Hfq is a multifunctional regulator in RNA metabolism of prokaryotes [4,5,36,37]. One of its functions is to modulate RNA polyadenylation catalyzed by PAP [14,15]. This enzyme is closely related to the CCA-adding enzyme that synthesizes and maintains the 3'-terminal CCA sequence on tRNAs. Due to sequence similarities, it is discussed that these enzymes share a common ancestor and interconverted into each other during evolution [16]. This fact led to the question whether the activity of these related enzymes is also modulated by identical proteins. Since Hfq has a well characterized stimulating effect on PAP, it was therefore investigated whether it also influences the activity of the CCA-adding enzyme.

Here, it is shown that Hfq is indeed stimulating the CCA-adding enzyme from *E. coli*. While it is well known that BSA has a non-specific stabilizing effect on enzyme preparations, leading also to reaction enhancement, the Hfq effect cannot be explained by simple protein stabilization alone, since it significantly exceeds the influence of BSA. Other RNA binding proteins are also much less efficient than Hfq in stimulating CCA-addition (comparable to BSA), which is a further indication that Hfq exerts a specific effect. Hence, PAP as well as CCA-adding enzyme are both stimulated by this protein in their activities. The stimulation of polyadenylation is based on interaction of Hfq with the RNA substrate, where it binds as a hexameric ring of identical subunits to A/U-rich single-stranded elements closely located to hairpin-like structures [2,7,11,38,39]. This effect on polyadenylation was also observed *in vivo*, where Hfq might also interact with PAP [15,40]. However, Hfq binding is not restricted to RNA transcripts carrying such structural recognition elements, but binds also to other, not yet identified sequences, as it was demonstrated for small regulatory RNA molecules and tRNAs, which is in agreement with our results [8]. In the case of tRNA precursor transcripts carrying leader and trailer sequences, Hfq seems to regulate tRNA maturation by slowing down RNase E-catalyzed cleavage, as both proteins recognize similar elements [8]. The tRNA transcripts, however, that were used as substrates for CCA-addition obviously do not carry typical Hfq binding motifs but belong to the pool of transcripts that interact with Hfq even in the absence of those elements. This tRNA – Hfq recognition is obviously mediated by amino acid residues of the protein that differ from those identified for interactions with mRNA or small regulatory RNAs. Indeed, replacement of V43 by R only affects stimulation of polyadenylation and interaction with mRNA [33], while substitution of K56 by A interferes with ncRNA stabilization [32]. Yet, both variants do not affect the enhancement of CCA-addition.

Interestingly, a strong Hfq-specific stimulation in CCA-addition was only observed at tRNA concentrations at 0.25 μM and above, while at lower concentration, the presence of BSA led to a comparable enhancement. Since in this experiment the tRNA concentration was increased, while the Hfq concentration remained constant, the Hfq-tRNA ratio was dropping from 19:1 to 0.75:1 (for the monomer; 3:1 to 0.13:1 for the hexamer). This led to a significant increase of the reaction rate (which is not observed with BSA). Therefore, it is possible that at low tRNA concentrations, a higher number of Hfq hexamers binds to the tRNA and blocks the interaction with the CCA-adding enzyme, forming an inactive Hfq/tRNA complex which might reduce the CCA incorporation to some degree. This interpretation is supported by the observation of supershifted bands (consisting of tRNA and Hfq) at increased concentrations of Hfq in gel shift experiments with radioactively labeled tRNA, indicating the binding of further Hfq molecules to the tRNA (data not shown). The presence of such an excess of Hfq can interfere with enzyme – substrate interaction and is likely to lead to an increase of apparent K_M _values. Such inhibiting Hfq complexes were also observed with the small regulatory RNA DsrA, where stoichiometry of Hfq interaction determines the translatability of *rpoS *mRNA [41]. At higher substrate tRNA concentrations, the inhibitory effect disappeared, presumably because less Hfq molecules bind to an individual tRNA, rendering it accessible for the CCA-adding enzyme.

How Hfq increases the reaction velocity but does not influence K_M _is not clear yet. Apparently, it exerts an indirect effect on the CCA-adding enzyme, since no supershift of tRNA, Hfq and CCA-adding enzyme was observed in the gel shift analysis (Fig. 2A), and the kinetic data indicate that the affinity of the CCA-adding enzyme to the tRNA is not affected by Hfq. The absence of such a supershifting complex shows that although such an interaction has to occur during the enhanced CCA-addition, it is not stable or persistent enough to be detected in the experiment. However, it is conceivable and in agreement with the absence of a supershift with CCA-adding enzyme that the Hfq/tRNA complex facilitates product release by weakening hydrogen bonds or ionic interactions between enzyme and tRNA. Subsequently, Hfq might also release the completed tRNA and cycle to a new substrate for CCA-addition. A similar dissociation of Hfq and RNA was already observed [41].

Taken together, it seems that Hfq is involved in the CCA-end restoration in *E. coli*, where about 15% of the tRNAs have a damaged 3'-end [42]. The abundance of 10.000 Hfq hexamers per cell is likely to be sufficient to enhance the repair of these truncated CCA-termini [35]. Additionally, there is growing evidence that Hfq-related Lsm-like proteins in other organisms are also participating in tRNA processing, as depletion of Lsm-like proteins in yeast led to the accumulation of several tRNA precursors [43]. In this case the Lsm proteins obviously do not compete for tRNA binding (as Hfq and RNase E), but seem to recruit other processing factors to enhance tRNA maturation. Nevertheless, these data indicate that Lsm-like proteins (including Hfq) might have a general role in the processing pathway of tRNA transcripts.

## Conclusion

The results reported here indicate that the multifunctional Lsm-like protein Hfq stimulates not only the activity of poly(A) polymerase, but also the reaction catalyzed by the CCA-adding enzyme, where it forms a complex with the tRNA substrate. The formation of this complex between Hfq and tRNA is particularly interesting, as it demonstrates a hitherto unknown specific and thermodynamically stable interaction of this protein with a mature tRNA transcript lacking the CCA-terminus. The kinetic analysis indicates that this complex does not affect the substrate affinity of the CCA-adding enzyme, but probably enhances the release of the reaction product, which is consistent with the fact that Hfq, tRNA and CCA-adding enzyme do not form a stable tertiary complex in the gel shift experiments.

Taken together, Hfq can stimulate the activity of the two closely related nucleotidyltransferases poly(A) polymerase and CCA-adding enzyme. It will be interesting to see whether – and how – this protein modulates also the activities of other members of this enzyme class.

## Methods

### RNA preparation

tRNA substrates (unlabeled or internally labeled with α^33^P-UTP) with or without CCA terminus or carrying parts of it (-C, -CC) were produced by *in vitro *transcription [44]. Transcripts were purified by denaturing polyacrylamide gel electrophoresis. Bands were cut out with a sterile blade, and tRNA was eluted by incubation in 0.5 M ammonium acetate (pH 5.7), 0.1 M EDTA, 1 mM MgCl_2 _and 0.1% (w/v) SDS at 4°C overnight. After ethanol precipitation, the transcript was dissolved in water, dephosphorylated [44] and purified by phenol-chloroform extraction.

### Protein expression and purification

CCA-adding enzyme and Hfq proteins from *E. coli *were overproduced and purified as described [18,33].

### *In vitro *activity assays

Up to 0.1 pmol CCA-adding enzyme were incubated in a total volume of 20 μl in the presence of NTPs (1 mM) and 5 pmol tRNA lacking a CCA terminus (internally labeled with α-^33^P-UTP) in 30 mM HEPES/KOH (pH 7.6), 30 mM KCl, 6 mM MgCl_2 _and 2 mM DTT at 30°C for varying incubation times. These assays contained 7.5 pmol Hfq or Hfq variants (calculated as monomer), 7.5 pmol BSA or 7.5 pmol RNA binding protein (HU, NusA, TGT, RNase P protein). After ethanol precipitation, reaction products were separated by denaturing gel electrophoresis and analyzed using a STORM 860 optical scanner and Image Quant software.

### Gel shift experiments

5'-^32^P-labeled tRNA was incubated in presence or absence of CCA-adding enzyme, Hfq, or BSA (or combinations as indicated) under conditions described above. In control experiments for Hfq binding, competitor RNA was added in increasing amounts (20 to 600 fmol) to the 5'-^32^P-labeled tRNA (20 fmol). After addition of 5 μl loading dye (50% glycerol, 0.1% bromophenol blue, 0.1% xylene cyanol), RNA/protein complexes were separated on a native 6% polyacrylamide gel and visualized by autoradiography.

For the control experiments, the following transcripts were used:

T7 polymerase run-off transcript of an EcoRV-linearized pCR Topo 2.1 plasmid:

5'-GGG CGA AUU GGG CCC UCU AGA UGC AUG CUC GAG CGG CCG CCA GUG UGA UGG AU-3'

T7 polymerase run-off transcript (118 nt), corresponding to the 3'-part of the *rpsO *mRNA, including a poly(A) tail of 18 residues, carrying an Hfq binding site [14].

*In vitro *tRNA transcripts with complete or partial CCA ends [44].

### Kinetic Analysis

For Michaelis-Menten kinetics, 0.1 pmol CCA-adding enzyme were tested in 20 μl reaction volumes with six data points in the reaction buffer (see above) containing 1 mM NTPs and 5 μCi α-^32^P-ATP (3000 Ci/mmol). Unlabeled tRNA^Phe ^was titrated between 0.1 μM and 2.5 μM. Assays were incubated at 30°C for 7 min in the presence or absence of 37.5 pmol Hfq (calculated for the monomer) in order to avoid an underrepresentation of Hfq/tRNA complexes at higher RNA concentrations. An identical concentration was used for BSA. The reaction was stopped by ethanol precipitation and products were separated by denaturing polyacrylamide gel electrophoresis. Densitometric analysis of reaction products was carried out using a STORM 860 phosphor imager and ImageQuant software. Apparent kinetic parameters of 5 independent experiments were determined using GraphPad Prism software.

## Authors' contributions

MS and SB performed all presented experiments, MS, HB and MM conceived the study and analyzed the data. MS, EH, HB and MM wrote the manuscript. All authors read and approved the final manuscript.
